# Smaller clinical trials for decision making; a case study to show p-values are costly

**DOI:** 10.12688/f1000research.15522.2

**Published:** 2018-09-27

**Authors:** Nicholas Graves, Adrian G. Barnett, Edward Burn, David Cook

**Affiliations:** 1 Institute of Health and Biomedical Innovation, Queensland University of Technology, Brisbane, QLD, 4059, Australia; 2 Nuffield Department of Orthopaedics, Oxford University, Oxford, OX3 7LD, UK; 3 Princess Alexandra Hospital, Brisbane, Brisbane, QLD, 4102, Australia

**Keywords:** decision making, RCT, sample size, waste in research

## Abstract

Background: Clinical trials might be larger than needed because arbitrary levels of statistical confidence are sought in the results. Traditional sample size calculations ignore the marginal value of the information collected for decision making. The statistical hypothesis testing objective is misaligned with the goal of generating information necessary for decision-making. The aim of the present study was to show that for a case study clinical trial designed to test a prior hypothesis against an arbitrary threshold of confidence more participants were recruited than needed to make a good decision about adoption.

Methods: We used data from a recent RCT powered for traditional rules of statistical significance. The data were also used for an economic analysis to show the intervention led to cost-savings and improved health outcomes. Adoption represented a sensible investment for decision-makers. We examined the effect of reducing the trial’s sample size on the results of the statistical hypothesis-testing analysis and the conclusions that would be drawn by decision-makers reading the economic analysis.

Results: As the sample size reduced it became more likely that the null hypothesis of no difference in the primary outcome between groups would fail to be rejected. For decision-makers reading the economic analysis, reducing the sample size had little effect on the conclusion about whether to adopt the intervention. There was always high probability the intervention reduced costs and improved health.

Conclusions: Decision makers managing health services are largely invariant to the sample size of the primary trial and the arbitrary p-value of 0.05. If the goal is to make a good decision about whether the intervention should be adopted widely, then that could have been achieved with a much smaller trial. It is plausible that hundreds of millions of research dollars are wasted each year recruiting more participants than required for RCTs.

## Introduction

Informed patients, thoughtful clinicians and rational health planners make decisions about the services and treatments provided using the best information available, and all decisions are made under conditions of uncertainty ^1, 2^. We examine a situation where sufficient evidence arises from a clinical trial to inform a decision about changing services before the conventional statistical stopping point for a clinical trial is reached. This paper is about the tension between the ‘precision’ and the ‘impact’ of a scientific measurement ^3^ and how that tension might dictate the sample size of a clinical trial.

Imagine a new treatment is compared against the best contemporary alternative in a well conducted randomised controlled trial (RCT). The design requires 800 participants in total based on a standard sample size calculation of 5% type 1 error and 80% power. The new treatment is more efficacious, prolongs life of high quality and saves more money than it costs to implement. The evidence to support these conclusions can be seen in the data after only 200 trial participants have been recruited, but primary outcomes are not yet statistically significant. Clinical equipoise, the cornerstone of ethical treatment allocation is lost, yet the conventions of hypothesis testing and arbitrary power calculation demand a further 600 participants are recruited. The information arising from the additional 600 participants is unlikely to change the actions of a rational decision maker who wishes to adopt the new treatment. Yet scarce research funds are used up meaning opportunities to fund other research are lost, and some patients have been consented and allocated to a treatment that we could not recommend, nor would we chose for ourselves or our families.

The utility of clinical trials for those managing health services and making clinical decisions is under debate and traditional paradigms are being challenged ^4^. The chief claim of this paper is that an RCT designed to test a hypothesis using traditional rules of inference might have more participants than required, if the goal is to make a good decision. Waste in research arises from routine use of arbitrary levels of statistical confidence ^5^ and because the trial data are considered in isolation ^6^. The marginal value of the information acquired for the purpose of making a good decision is not made explicit. Important information for the purpose of decision making often lies outside the clinical trial process. The plausibility of our claim is demonstrated by re-analysing a recent RCT ^7^.

### Choosing a sample size for hypothesis testing

For the design of superiority trial, the aim is to have a high likelihood of sufficient evidence to confidently reject a null hypothesis that two treatments are equivalent when treatments differ by a specified difference. This difference is usually based on either clinical importance or a best guess of the true treatment effect. Inference based on this approach has two types of potential errors. A false-positive or type I error of rejecting the null hypothesis when there is no difference, with probability α. A false negative or type II error of not rejecting the null hypothesis when there is an effect, with probability β. The sample size of the trial is calculated to give an acceptable type I error rate and power (1–β), typically 0.05 for α and 0.8 to 0.9 for the power. The final analysis summarises the incompatibility between the data and the null hypothesis ^8^. If the p-value is below the standard 5% limit the null hypothesis of no effect is rejected. A ‘statistically significant’ result is then celebrated and typically used to support a decision to make a change to health services.

### Choosing a sample size for decision making

We assume the objective of decision-makers who manages health services is to improve outcomes for the populations they serve. Because this challenge will be addressed with finite resources not every service or new technology can be made available for a population. Decision-makers therefore require knowledge of the health foregone from not funding services displaced by the services that are funded ^9^. The services that are provided should generate more health benefits per dollar of cost when compared to those that are not. With this criterion satisfied the opportunity cost from the services not provided is minimised. A rational decision maker will logically follow these rules: do not adopt programmes that worsen health outcomes and increase cost; adopt programmes that improve health outcomes and decrease costs; and, when they face a situation of increased cost for increased health outcomes they prioritise programmes that provide additional health benefits for the lowest extra cost ^10^. They will continue choosing cost-effective services until available health budgets are exhausted. An appropriate and generic measure of health benefit is the quality adjusted life year (QALY) ^11^. While this approach does not consider how health benefits are distributed among the population there is a framework for including health inequalities in the economic assessment of health care programmes ^12^.

In choosing a sample size for a clinical trial to evaluate a new service or technology a decision-maker will consider the uncertainty in the conclusion about how costs and health benefits change by adoption. The aim is to reduce the likelihood of making the wrong decision. They will make rational and good decisions, and they will manage uncertainty rather than demand an arbitrarily high probability of rejecting a null hypothesis. Methods are available to estimate the expected value of information and so the optimal sample size for a trial is dependent on the context specific costs and benefits of acquiring extra information ^13^. Each decision is context dependent and the ‘one size fits all’ approach to sample size calculation is arbitrary and potentially wasteful. This holistic approach should be a priority for designing, monitoring and analysing clinical trials.

## Methods

### The TEXT ME RCT: A case study

A case study to illustrate the differing evidential requirements of the ‘hypothesis-testing’ and ‘decision-making’ approaches is provided by the RCT of the Tobacco, Exercise and Diet Messages (TEXT ME) intervention ^14^. This health services program targeted multiple influential risk factors in patients with coronary heart disease, with SMS text messages. Advice and motivation was provided to improve health behaviours and it was supplementary to usual care. The hypothesis was that the intervention would lower plasma low-density lipoprotein cholesterol by 4.5 mg/dL at 6 months for participants compared with those receiving usual care ^15^. The required sample size was 704 participants for 90% power ^15^ and the trial recruited and randomised 710 participants ^7^. The mean difference between the intervention and control group was –5 mg/dL, (95% CI –9 to 0 mg/dL). With a p-value of 0.04, the null hypothesis was rejected. Evidence for health effects were also sought on other biomedical and behavioural risk factors, quality of life, primary care use and re-hospitalisations. Clinically and statistically significant effects were also found for systolic blood pressure (mean difference –8 mmHg, p<0.001), body mass index (–1.3 kg/m ^2^, p<0.001) and current smoking (relative risk of 0.61, p<0.001).

The TEXT ME trial data were used to inform an economic evaluation of the potential change to costs and health benefits measured in quality adjusted life years to the community from a decision to adopt the programme ^16^. The observed differences in low-density lipoprotein cholesterol, systolic blood pressure and smoking were combined with reliable external epidemiological evidence to estimate the reduction in acute coronary events, myocardial infarction and stroke and were extrapolated over the patients expected remaining life times. The costs of providing the intervention, the projected costs of the treatment of acute events and general primary care use and expected mortality were all informed by data sources external to the primary trial ^16^. The findings revealed that TEXT ME was certainly going to lead to better health outcomes and cost savings. The conclusion was that a rational decision-maker should fund and implement the TEXT ME program. Once available an informed clinician would then recommend TEXT ME to coronary patients, and enough patients would sign up to create benefits for individuals and the health system. Using the TEXT ME study, we consider whether the same decision could have been made at an earlier stage with fewer participants enrolled in the primary trial.

### Data analysis

We examine the effect of a reduced sample size on the results of both the hypothesis-testing analysis for differences in low-density lipoprotein cholesterol, and the economic evaluation of the intervention. From the original 710 participants, smaller samples between 100 and 700 patients in increments of 100 were considered with the resampling done with replacement. The ‘p-value’ and ‘economic’ analyses were re-run using the data provided by the randomly selected patients and this process was repeated 500 times for each sample size. The simulations and figures were created using R (version 3.1.0). The code is available on GitHub https://github.com/agbarnett/smaller.trials but we are unable to share the primary data from the TEXT ME RCT.

### Counter-example of no treatment effect

To illustrate this approach with treatments that are equally effective, we used the same methods as above, but created data using the TEXT ME trial where the two groups had equivalent outcomes. We did this by randomly allocating patients to the TEXT ME intervention or usual care, and then resampling with replacement to create a new version of the study sample. We assumed there was no risk reduction for the TEXT ME group, and used the same uncertainty in risk reduction as per the previous model.

## Results

**Figure 1.**
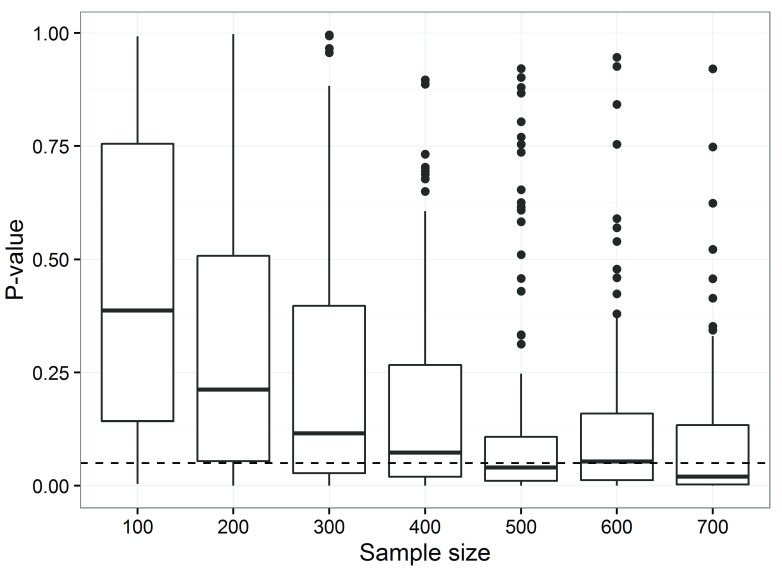
P-values increase as sample sizes decrease for the observed differences in low-density lipoprotein cholesterol (based on 500 simulations per sample size). The dotted horizontal line is the standard 5% threshold. The boxes are the 25th and 75th percentiles with the median as the central line. The upper whisker extends from the third quartile to the largest value no further than 1.5 * IQR from the quartile (where IQR is the inter-quartile range). The lower whisker extends from the 1st quartile to the smallest value at most 1.5 * IQR of the quartile. Data beyond the end of the whiskers are called ‘outlying’ points and are plotted individually.

The effect of reducing the sample size for hypothesis-testing objectives was to simulate studies that traditional hypothesis testing approaches would deem underpowered, see Figure 1.

Only for a sample size of 500 participants or more would the majority of trials find a statistically significant difference in average low-density lipoprotein cholesterol between groups ( Figure 1). Even at a sample size of 700 around 30% of trials would be expected to make the ‘wrong’ inference of not rejecting the null hypothesis. This is consistent with a priori analytic estimates of sample size to address the hypothesis.

**Figure 2.**
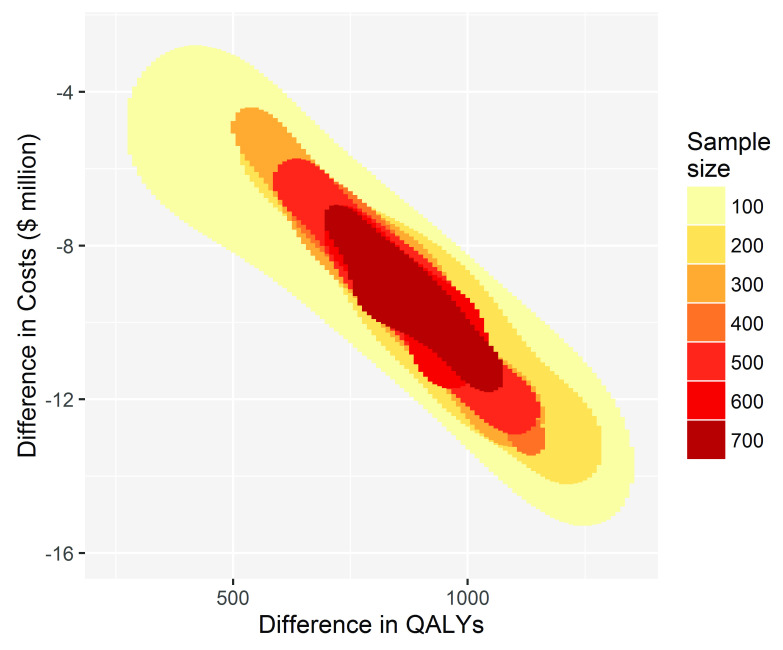
The conclusion for decision-making becomes more uncertain but does not change with decreasing sample size. The x-axis shows the QALY gains for TEXT ME over usual care, and the y-axis shows the cost savings.

To inform decision making using cost-effectiveness as the criterion, reducing the sample size has little effect on the conclusion of whether to fund, recommend and participate in TEXT ME, see Figure 2. For every simulation for each sample size the decision to adopt TEXT ME led to cost savings shown on the y-axis and gains to health, measured by QALYs shown on the x-axis.

A sample size of 100 or more in the primary trial would convince a risk neutral and rational decision maker that TEXT ME is both cost-saving and health improving, and so should be adopted. The imprecision surrounding this inference increases as the sample size reduces, but the decision-making inference does not change. If the goal is to make a good decision about whether TEXT ME should be adopted widely, then that could have been achieved with a much smaller trial, one that enrolled as few as 100 patients. This would have been a cheaper and quicker research project releasing scarce research dollars for other important projects.

**Figure 3.**
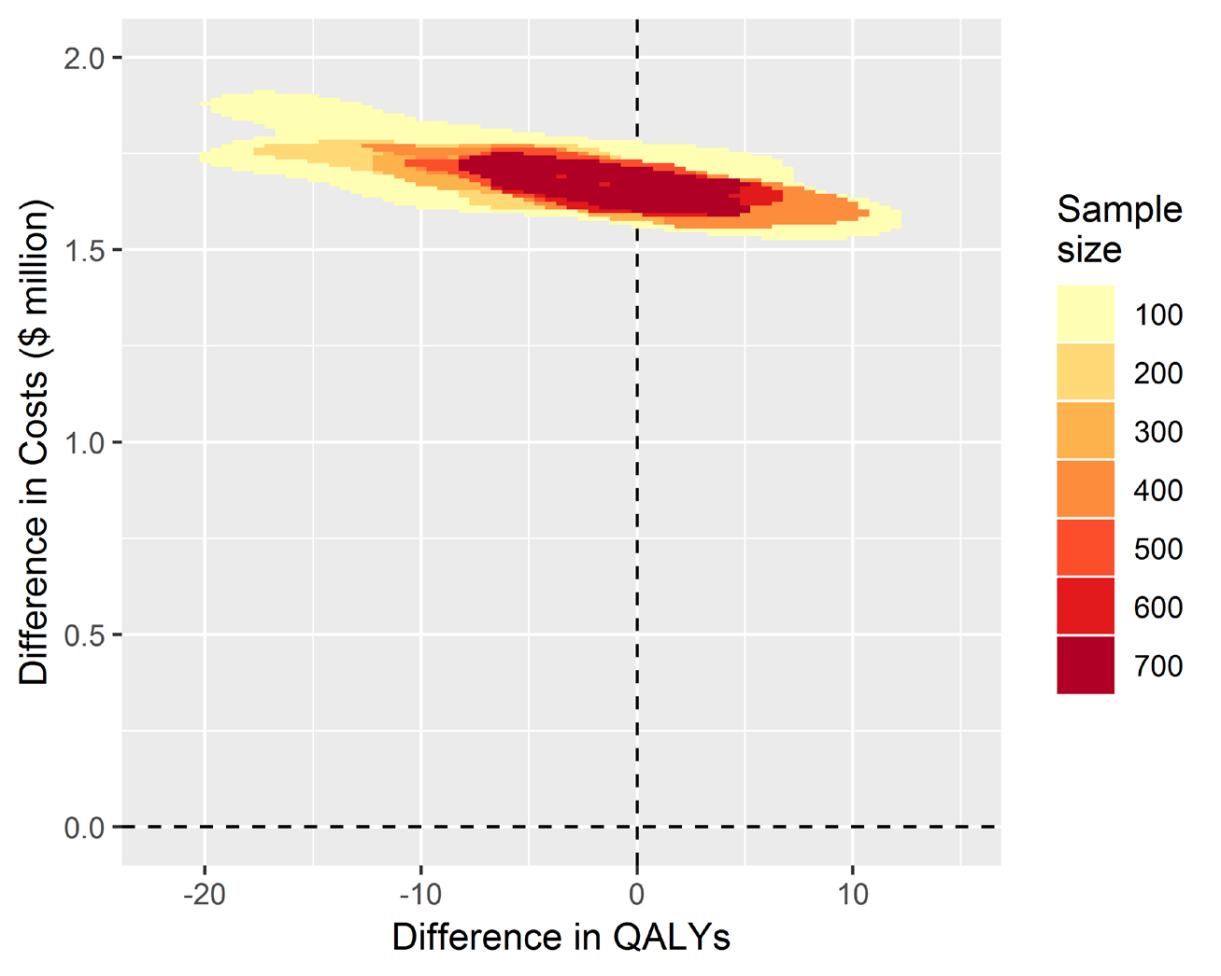
The conclusion for decision-making is clear when there is no treatment effect, costs are increased for no change to health benefits for all sample sizes.

When we simulated studies where there was no treatment effect, all the costs of implementing the TEXT ME program of around 1.5 million dollars for the cohort of 50,000 patients were incurred, but none of the health benefits and associated cost savings were realised. The estimates of change to health benefits straddled the zero line with a spread covering a relatively small change in QALYs of around 20 lost to 12 gained. The inference for decision makers is clear at any sample size that adoption would be a poor decision ( Figure 3).

Data used for a simulation of Figure 2 Click here for additional data file. http://dx.doi.org/10.5256/f1000research.15522.d212377
Copyright: © 2018 Graves N et al. 2018 Data associated with the article are available under the terms of the Creative Commons Zero "No rights reserved" data waiver (CC0 1.0 Public domain dedication).

## Discussion

RCTs have become “massive bureaucratic and corporate enterprises, demanding costly infrastructure for research design, patient care, record keeping, ethical review, and statistical analysis” ^17^. A single phase 3 RCT could today cost $30 million or more ^18^ and take several years from inception to finalisation. These trials are powered for arbitrary rules of statistical significance. Critics of this approach ^3^ argue “that some of the sciences have made a mistake, by basing decisions on statistical significance” and that “in daily use it produces unchecked a loss of jobs, justice, profit, and even life”. The mistake made by the so called ‘sizeless scientist’ is to favour ‘Precision’ over ‘Oomph’. A ‘sizeless scientist’ is more interested in how precisely an outcome is estimated and less interested in the size of the implications for society or health services of any observed change in the outcome. They do not appear interested in the facts that “significant does not mean important and insignificant does not mean unimportant”. Even experts in statistics have been shown to interpret evidence poorly, based on whether the p-value crosses the threshold of 5% for statistical significance ^19^.

Researchers today are calling for a shift towards research designed for decision making ^20^. Yet this is not new, in 1967 Schwartz & Lellouch ^21^ made a distinction between ‘explanatory’ and ‘pragmatic’ approaches. The former seeks ‘proof’ of the efficacy of a new treatment and the latter is about ‘choosing’ the best from two treatments. Patients, clinicians and payers of health care are interested in whether some novel treatment or health programme should be adopted over the alternatives.

There are many choices to be evaluated and many useful clinical trials to be undertaken, yet research budgets to support these are insufficient ^22^. Funding a larger number of smaller trials to enable correct decisions about how to organise health services more frequently is a sensible goal. A hypothesis-testing approach maintains that a uniform level of certainty around these decisions is desirable, and needed by all stakeholders: managers, clinicians and patients. Yet the costs and benefits of every decision made are context-specific. Striving to eliminate uncertainty is likely to be inefficient use of research funding, where the benefit of achieving a given level of certainty is low or the prescribed precision unnecessary. We are not the only group that are advocating for this approach, and others have used cost-effectiveness as a criteria for dynamically deciding the necessary size of an ongoing trial ^23^. There is a wider literature on decision making including economic data. Decision-making should address the costs and benefits throughout the life cycle of an intervention ^24^, with consideration of whether decisions could be made based on current evidence and whether additional research needs to be undertaken ^25^. Other considerations for decision making under conditions of uncertainty have been established and reviewed in detail ^26^.

Our observations contradict advice by Nagendran *et al.* ^27^ who suggest researchers aim to “conduct studies that are larger and properly powered to detect modest effects”. This approach promotes using p-values for decision making without a more encompassing evaluation of all outcomes that are relevant for decision-making.

We suggest the decision making approach to sample size calculation would often lead to smaller trials, but not always. If rare adverse events had a substantial impact on cost and health outcomes the trial may be larger than a hypothesis testing trial powered for a single outcome, which was not the adverse event. This may especially be the case for trials of new drugs. There are some good arguments against smaller trials. A large trial with lots of data might help future proof an adoption decision. If costs, frequencies of adverse events or baseline risks change over time then a large trial might render sufficient information to defend the adoption decision in the future as compared to a small trial. There might also not be another opportunity to run an RCT, for ethical or funding reasons, and so gathering a lot of data when the chance arises could be wise. Smaller trials, despite being well designed, might find a positive result that overestimates the real effect ^28^. This may have happened with our example of TEXT ME and a more conservative estimate of the intervention effect would likely come from a meta-analysis or repeated trial. Indeed Prasad *et al*. ^29^ found from 2,044 articles published over 10 years in a leading medical journal, 1,344 were about a medical practice, 363 of them tested an established medical practice and for 146 (40%) the finding was that practice was no better or worse than the comparator implying a reversal of practice. Those who deliver health services are unlikely to be rational and risk neutral. There is often scepticism and inertia when a change to practice is suggested and some clinicians will only change when evidence is overwhelming. Lau *et al*. ^30^ did a cumulative meta-analysis of intravenous streptokinase for acute myocardial infarction with mortality as the primary outcome. They showed the probability the treatment reduced mortality was greater than 97.5% by 1973 after 2,432 patients had been enrolled in eight trials. By 1977, after 4,084 patients had been enrolled in thirteen trials the probability the treatment was effective was more than 99.5%. By 1988, 36 trials had been completed with 36,974 patients included confirming the previous conclusion.

Our case study demonstrates - for a single carefully conducted trial - that more information might have been collected than was necessary for a good decision to be made about a decision to adopt the intervention. We did not cherry pick this trial, but selected it because it was a recent economic analysis and had broad implications for health. The differences in necessary sample sizes and evidence will depend on context and design of trials. It might often be that smaller and so faster and cheaper trials are sufficient for good decision-making. This would release scarce research dollars that funding bodies could use for other valuable projects. Our approach is part of the drive toward increasing the value of health and medical research, which currently has a poor return with an estimated 85% of investment wasted ^31^. Further, as adaptive trials gain traction, decision based designs provide flexibility, facilitating faster evolution of implementable findings.

## Data availability

The data referenced by this article are under copyright with the following copyright statement: Copyright: ï¿½ 2018 Graves N et al.

Data associated with the article are available under the terms of the Creative Commons Zero "No rights reserved" data waiver (CC0 1.0 Public domain dedication). 

The datasets used and/or analysed for the TEXT ME trial are not publicly available due to data sharing not being approved by the local ethics committee. To access the data, the corresponding author of the primary trial should be contacted ( cchow@georgeinstitute.org.au).

A random sample of the TEXT ME clinical trial data that has similar features to the TEXT ME data is provided in the code used to create the simulations and figures, which is available on GitHub: https://github.com/agbarnett/trials.smaller

Archived code as at time of publication: http://doi.org/10.5281/zenodo.1322459 ^32^

Dataset 1: Data used for a simulation of Figure 2. DOI, 10.5256/f1000research.15522.d212377 ^33^
