# Supplementary material for: Smaller clinical trials for decision making; a case study to show p-values are costly
Source: F1000Res. 2018 Sep 27;7:1176. Originally published 2018 Aug 2. [Version 2] doi: 10.12688/f1000research.15522.2 (PMC9555245; doi:10.12688/f1000research.15522.2)
Supplement: Peer review history

# Peer review: Reviewer response for version 2

**DOI:** 10.5256/f1000research.17883.r151315

**Published:** 2022-10-11

**Authors:** Daniel Benjamin Mark

**Affiliations:** 1 Division of Cardiology, Department of Medicine, Duke University Medical Center, Durham, NC, USA

This article addresses an important and provocative idea, namely that we are making our clinical trials larger and more expensive than is necessary for rational decision making. I suspect the authors are correct but would offer several general reasons why I think the case they have presented overlooks some key elements that help explain why things are less than optimally efficient in the sense proposed by the authors. 

 First, the trial they use to illustrate their ideas is a relatively simple test of text messaging to improve risk factor management. The intervention was not expensive and did not involve any complex risk benefit considerations. However, most of the trials that affect clinical management and that have a large health care budgetary impact involve interventions that are very expensive (with the potential to generate annual expenditures in the US of multiple billions of dollars a year) and often involve complex issues of risk and benefits. Regulatory approval in the US has typically involved many smaller trials leading up to two pivotal phase III trials that generally both need to be "positive" on their primary endpoint, meaning they need to show the treatment effect has a p< 0.05. Failing to achieve the requisite level of "evidence" in this context will typically result in a decision not to grant regulatory approval, in which case the company developing the therapy is faced with the decision about whether to abandon the work and investment to that point or to invest more in what may be an even larger and more expensive next trial. Regulators in the US at least currently are charged with ensuring treatments are both safe and effective before granting market approval and they do not have any responsibility for addressing cost effectiveness or budget impact. So these decision makers at least would not accept a lower standard of evidence in order to improve efficiency and the level of evidence has a big effect on their decision making. 

 Second, clinical practice guideline committees and major clinical journals also triage clinical trial interpretation according to strict statistical significance criteria. Many trials published in journals such as NEJM have had the tested therapy declared ineffective because of a p value in the range of 0.06 to 0.10. In such situations, guideline committees are very likely to accept the official interpretation and make recommendations accordingly. These decision makers also do not have any interest in accepting lower levels of precision in order to improve the efficiency of the health care system. Part of the difficulty here is the (mis)understanding of what significance tests/p values can and cannot tell us about the outcomes of a clinical experiment/RCT, as the authors discuss. Fixing that by getting clinicians and statisticians and guideline committees to use more flexible, comprehensive interpretive approaches to evidence has proven quite difficult. 

 Third, the authors describe their target audience as health care decision makers managing health care services. The assumptions of economics regarding the decisions being made by rational decision makers who have the goal of maximizing the health benefits for the largest number of their population possible is an interesting model but it's not clear that it describes any actual health care system and how it functions. In the US, the primary focus is on budget impact not efficiency. In countries where economic analysis is required for reimbursement, budget impact still seems to be a dominant consideration.  

 The implications for the paper would seem to be that the authors should consider in their presentation a bit more about what the needed infrastructure is in order for their recommendations to be adopted. What kind of trials would be suitable for smaller tests accepting less precision in exchange for more rapid efficiency and lower costs? One area might be implementation trials, in other words, trials testing the deployment into the practice of things we already know work from prior, large pivotal trials. The example provided seems to fit in this category. 

 The global nature of scientific medicine means that the knowledge about effective but expensive therapies is widely available to providers and patients across health systems. Health systems can therefore no longer refuse to provide their citizens with advances simply because of the expense, but at the same time they have to control the growth of health care spending. Making more therapies available with a greater uncertainty accepted in the estimated treatment effect may be difficult to sell as a general concept.  One could, I suppose, argue that if regulatory approval were not so expensive, companies would not need to charge such high prices for new advances. Not clear that there is any appetite among health regulators for moves in this direction and even if some countries did adopt such a program, the developers of new therapies facing the global market forces might not benefit enough to alter pricing. 

 Besides some forms of implementation research, I think what the authors are proposing could work in the context of "evidence free zones", areas of medicine where there is little beyond anecdote and expert opinion to use in decision making. In that context, an inexpensive trial that provides some reliable evidence, accepting a higher level of uncertainty, can still be used to change/guide practice and policy. Despite all the trials that get reported every year, much of current practice guidelines still consist of expert opinion, as large clinical trials are only done in select areas where funding sources exist willing to support the work. Most of clinical medicine falls outside these zones.  

 In summary, I think the authors have raised an important and interesting issue. I would ask them to consider discussing a bit more of the real world nuances of how and where this might work and where it is unlikely to be accepted.

Is the work clearly and accurately presented and does it cite the current literature?

Yes

If applicable, is the statistical analysis and its interpretation appropriate?

Partly

Are all the source data underlying the results available to ensure full reproducibility?

Yes

Is the study design appropriate and is the work technically sound?

Partly

Are the conclusions drawn adequately supported by the results?

Partly

Are sufficient details of methods and analysis provided to allow replication by others?

Yes

Reviewer Expertise:

Clinical trials, outcomes research, clinical economics, cardiovascular medicine

I confirm that I have read this submission and believe that I have an appropriate level of expertise to confirm that it is of an acceptable scientific standard, however I have significant reservations, as outlined above.

# Peer review: Reviewer response for version 2

**DOI:** 10.5256/f1000research.17883.r46419

**Published:** 2019-09-09

**Authors:** Steven A Julious

**Affiliations:** 1 Medical Statistics Group, University of Sheffield, Sheffield, UK

While I agree with the sentiments of the authors that there can be instances where a conventional sample size calculation may not be appropriate. I do have an issue with the generalisations they are making from a single trial to studies costing $30m.

 The gold standard for an assessment of efficacy right or wrong is a formal hypothesis test. There is a need to definitively show evidence of clinical effect for most interventions.

 The example that was quoted in the paper is a cheap intervention. Low cost interventions may be anticipated to observed small effects: so small that the expense of undertaking a clinical trial could be prohibitively expensive.

 I have recent experience of two trials investigating low cost interventions in the U@UNI trial and the PLEASANT trial. ^1^^,^^2^^,^^3^^,^^4^ In both these trials the interventions could be shown to be cost effective (or cost saving). It could be contended in both the sample size could have been based on cost effectiveness.

 In summary, therefore, there is merit in what the authors are suggesting but there needs to be consideration as to when the arguments could be applied.

Is the work clearly and accurately presented and does it cite the current literature?

Yes

If applicable, is the statistical analysis and its interpretation appropriate?

No

Are all the source data underlying the results available to ensure full reproducibility?

Yes

Is the study design appropriate and is the work technically sound?

Yes

Are the conclusions drawn adequately supported by the results?

No

Are sufficient details of methods and analysis provided to allow replication by others?

Yes

Reviewer Expertise:

Medical statsitics, clinical trials

I confirm that I have read this submission and believe that I have an appropriate level of expertise to confirm that it is of an acceptable scientific standard.

## References

[1] : The cost-effectiveness of an updated theory-based online health behavior intervention for new university students: U@Uni2. *Journal of Public Health and Epidemiology* .2016;8(10) : 10.5897/JPHE2016.0833 191-203 10.5897/JPHE2016.0833

[2] : A theory-based online health behaviour intervention for new university students (U@Uni): results from a randomised controlled trial. *BMC Public Health* .2014;14: 10.1186/1471-2458-14-563 563 10.1186/1471-2458-14-563 24903620PMC4067627

[3] : PLEASANT: Preventing and Lessening Exacerbations of Asthma in School-age children Associated with a New Term - a cluster randomised controlled trial and economic evaluation. *Health Technol Assess* .20(93) : 10.3310/hta20930 1-154 10.3310/hta20930 28005003PMC5220260

[4] : Economic Evaluations Alongside Efficient Study Designs Using Large Observational Datasets: the PLEASANT Trial Case Study. *Pharmacoeconomics* .2017;35(5) : 10.1007/s40273-016-0484-y 561-573 10.1007/s40273-016-0484-y 28110382PMC5385191

# Peer review: Reviewer response for version 2

**DOI:** 10.5256/f1000research.17883.r38819

**Published:** 2018-10-09

**Authors:** Stephen Senn

**Affiliations:** 1 Competence Center for Methodology and Statistics, Luxembourg Institute Of Health, Strassen, Luxembourg; 2 School of Health and Related Research, University of Sheffield, Sheffield, UK

First let me apologise for a stupid slip in my first review. I referred to sampling *without *replacement and the authors quite rightly corrected me and pointed out that they sampled *with *replacement. That is, in fact, what I meant to say.

 Second, let me acknowledge that the authors have gone some way to answering my criticisms. However, I am not completely satisfied, so before changing my overall judgement of the paper, I am going to explain the problem again.

 Consider an alternative to sampling with replacement that will produce almost the same results they did. This is to compare a super trial in which the values for every patient in the TEXT ME trial are copied a huge number of times to create a million versions of each patient. Thus every patient in the trial has 999,999 identical virtual siblings. Where the TEXT ME trial had 710 patients we now have 710 million patients. Let us call this trial MegaText. If all these patients were real, then in the huge population of MegaText the effect seen would be the true effect. Now we can actually sample without replacement from MegaText and the result will be almost identical to sampling with replacement from the TEXT ME trial. The only slight difference is that in sampling without replacement from MegaText once a patient has been chosen there is a very slightly reduced chance of the patient being chosen again but since there are so many copies, this hardly matters.

 Therefore, sampling from the TEXT ME trial is almost identical to sampling from the MegaText trial and it thus follows that we are sampling from a population in which there is a genuine treatment effect and there is no uncertainty about this. Thus the right decision is known to be to implement the program. Of course, and their simulation shows this, if the trial is small enough, you will sometimes choose the wrong treatment. If the purpose of the trial is pragmatic in the sense of Schwartz and Lellouch(Schwartz, D. & Lellouch, J., 1967), then it is this Type III error that has to be guarded against.

 However, this raises the second issue. The decision to always choose what appears to be the better of two treatments being compared, however weak the evidence, is logical *if no further evidence can be obtained.* However, it is not necessarily logical if more information can be obtained at a modest cost. To see this, consider the case where a new treatment *N* is being compared to a standard treatment *S* and high values are good, as would be the case if we are measuring utility. The Bayesian posterior distribution for difference in effects ( *N-S*) is mainly in the positive area: it is more probable than not, taking all things into account that it is better to use *N*. However, there is a non-negligible probability that actually S is better. If information can be obtained at low cost it may be worth doing so just to exclude the possibility that S is after all better.

 Thus, I have some unease that the combination of starting with a proven treatment and showing that if one had carried out a smaller trial one would often come to an apparently good choice of treatment if one had to be made is quite as relevant to the practical problem that choosing a sample size is meant to solve. This is not to say that common approaches to doing this are good: far from it. They ignore the dimension of cost and this cannot be rationale.

 Thus, if what the authors wish to say is: “just because a trial has not found a significant result it does not follow that it cannot be used to decide to implement a new treatment if no further information will be forthcoming” there are some circumstances under which I could agree. If they wish to imply that standards should generally be less stringent than they currently are, I do not think this sort of investigation is particularly relevant.

Is the work clearly and accurately presented and does it cite the current literature?

Partly

If applicable, is the statistical analysis and its interpretation appropriate?

No

Are all the source data underlying the results available to ensure full reproducibility?

Yes

Is the study design appropriate and is the work technically sound?

Partly

Are the conclusions drawn adequately supported by the results?

No

Are sufficient details of methods and analysis provided to allow replication by others?

Partly

Reviewer Expertise:

I am a medical statistician with many years experience in dealing with problems associated with drug development and regulation.

I confirm that I have read this submission and believe that I have an appropriate level of expertise to confirm that it is of an acceptable scientific standard, however I have significant reservations, as outlined above.

## References

[1] : Explanatory and pragmatic attitudes in therapeutical trials. *J Chronic Dis* .1967;20(8) :637-48 486035210.1016/0021-9681(67)90041-0

# Response

**Published:** 2019-10-31

**Authors:** Nicholas Graves

**Affiliations:** Duke NUS Graduate Medical School, Singapore

We thank the reviewer for the additional comments and try to respond to the key issue that arose in the first review.

The reviewer says “ *simulating only from the case where the intervention is beneficial is not adequate*”, but we simulate because we are not certain the intervention is beneficial. Hence, we simulate from the observed data in order to see whether the intervention is beneficial on a meaningful scale.

In general, these simulations use multiple outcomes, some of which may have a positive mean (e.g., improvement in blood pressure and the associated health benefits/ health utility) and some have a negative mean (e.g., increase in costs). Hence, it is a composite estimate that is more complex that just one mean being positive or not.

We used this approach for another important question about whether a hand hygiene campaign should be funded and showed the conclusions varied for different states and territories of Australia, see Table 3 of this paper…

https://journals.plos.org/plosone/article?id=10.1371/journal.pone.0148190 

In South Australian, Tasmania & Western Australia the positive difference in the main outcome was not large enough to justify a decision to continue the campaign. But in Queensland, ACT and New South Wales we concluded the opposite.

We are not simply reliant on a single positive mean, which should happen for around 50% of studies where the intervention has no effect and hence is quite easy to achieve. Instead, we are looking at whether the observed difference is meaningful using the observed variation in the sample.

It is possible to use scenario analysis to simulate results under more pessimistic scenarios, such as a null treatment effect but reduction in costs, and these can be informative for decision makers.

# Peer review: Reviewer response for version 1

**DOI:** 10.5256/f1000research.16927.r37678

**Published:** 2018-09-07

**Authors:** Stephen Senn

**Affiliations:** 1 Competence Center for Methodology and Statistics, Luxembourg Institute Of Health, Strassen, Luxembourg; 2 School of Health and Related Research, University of Sheffield, Sheffield, UK

The authors propose that when a clinical trial is sought to inform practical decision-making, conventional standards of 'proof' may be too stringent and in consequence resources may be wasted. They illustrate this by simulating from a particular clinical trial, the TEXT ME trial, using progressively smaller sample sizes and suggest that a useful decision could have been made with fewer patients.

 The general argument presented is interesting and the conclusion that trials are sometimes too big if practical decision making is the object may well be correct. In this respect, a key distinction was made just over 50 years ago by Schwartz and Lelouch ^1^ between what they called *explanatory* or *pragmatic* approaches. In the former case 'proof' of the efficacy of a new treatment may be sought. In the latter case one may simply wish to choose the (plausibly) better of two treatments.

 However, unless I have misunderstood what the authors are doing (which I do not exclude but in that case they should clarify this) the simulation is not a valid proof of what they claim, even for the example chosen.

 The problem is the following. By simulating from the particular trial results, they are simulating from a universe in which the treatment is effective. This would be true even if the results from the TEXT ME trial had not been 'significant'. It is true of any trial in which the observed results favour the intervention. To see this consider that valid statistical analyses will typically have type I error rates in excess of a chosen nominal value if the mean under the intervention is greater (assuming high values are good) than the mean in the control group in the population in question. Provided that the type I error rate is controlled when this is not the case, this is a desirable property of such tests.

 Usually, the population in question is taken to be the population of all possible randomisations of the patients. Here, the authors sampled without replacement from the population. The population from which they are sampling is the population of results in the full TEXT ME trial. However, this is a population in which on average the results were better for the intervention.

 Hindsight is an exact science but those making practical healthcare decisions are involved in the quite different game of foresight and they need to know whether the decision they are about to make is a reasonable one. This requires their allowing for the possibility that the intervention is useless or even harmful. Thus a mixture of possible situations has to be considered: simulating only from the case where the intervention is beneficial is not adequate.

 In fact the precise nature of the mixture envisaged can have a huge effect on the inferences. Recently, a number of authors have called for statistical standards of evidence to be modified in the opposite direction. For instance Benjamin *et al.* ^2^  have suggested that the standard of p=0.005 should be adopted. David Colquhoun ^3^ has proposed an even more stringent standard of P=0.001.  This flows from the particular approach to Bayesian hypothesis testing which places a lump of probability on no difference between treatments. (See my blog ^4^ for a discussion.) In my opinion, these are not good suggestions for a number of reasons, including that such prior distributions are far too informative and that these authors implicitly assume, which is far from obviously the case, that the explanatory purpose of clinical trials is more important than the pragmatic one.

 However, I agree entirely with the authors, that as soon as practical decision-making involving economics is involved, it is the value of information that is important. In this connection, I can recommend the work of Forster, Pertile and colleagues ^5^^,^^6^. See also Burman *et al*. ^7^

 Thus, I think to make good their claim, the authors would, at the very least, need to simulate from a universe in which the intervention was not necessarily better than the control. Unless I have misunderstood, this was not the simulation they undertook.

Is the work clearly and accurately presented and does it cite the current literature?

Partly

If applicable, is the statistical analysis and its interpretation appropriate?

No

Are all the source data underlying the results available to ensure full reproducibility?

Yes

Is the study design appropriate and is the work technically sound?

Partly

Are the conclusions drawn adequately supported by the results?

No

Are sufficient details of methods and analysis provided to allow replication by others?

Partly

Reviewer Expertise:

I am a medical statistician with many years experience in dealing with problems associated with drug development and regulation.

I confirm that I have read this submission and believe that I have an appropriate level of expertise to confirm that it is of an acceptable scientific standard, however I have significant reservations, as outlined above.

## References

[1] : Explanatory and pragmatic attitudes in therapeutical trials. *J Chronic Dis* .1967;20(8) :637-48 486035210.1016/0021-9681(67)90041-0

[2] : Redefine statistical significance. *Nature Human Behaviour* .2018;2(1) : 10.1038/s41562-017-0189-z 6-10 10.1038/s41562-017-0189-z 30980045

[3] : An investigation of the false discovery rate and the misinterpretation of p-values. *R Soc Open Sci* .2014;1(3) : 10.1098/rsos.140216 140216 10.1098/rsos.140216 26064558PMC4448847

[4] : Double Jeopardy: Judge Jeffreys upholds the law.2015; Reference source

[5] : Optimal Bayesian sequential sampling rules for the economic evaluation of health technologies. *Journal of the Royal Statistical Society: Series A (Statistics in Society)* .2014;177(2) : 10.1111/rssa.12025 419-438 10.1111/rssa.12025

[6] : Late-stage pharmaceutical R&D and pricing policies under two-stage regulation. *J Health Econ* .2016;50: 10.1016/j.jhealeco.2016.06.002 298-311 10.1016/j.jhealeco.2016.06.002 27776744

[7] : Decision Analysis in Drug Development. In: Dmitrienko A, Chuang-Stein C, Agostino R, eds. Pharmaceutical Statistics Using SAS: A Practical Guide. *Cary: SAS Institute* .2007;385-428

# Response

**Published:** 2018-09-18

**Authors:** Nicholas Graves

**Affiliations:** Duke NUS Graduate Medical School, Singapore

The authors propose that when a clinical trial is sought to inform practical decision-making, conventional standards of 'proof' may be too stringent and in consequence resources may be wasted. They illustrate this by simulating from a particular clinical trial, the TEXT ME trial, using progressively smaller sample sizes and suggest that a useful decision could have been made with fewer patients.

 The general argument presented is interesting and the conclusion that trials are sometimes too big if practical decision making is the object may well be correct. In this respect, a key distinction was made just over 50 years ago by Schwartz and Lelouch1 between what they called explanatory or pragmatic approaches. In the former case 'proof' of the efficacy of a new treatment may be sought. In the latter case one may simply wish to choose the (plausibly) better of two treatments.

 RESPONSE: Thanks for flagging this interesting paper on trials and pragmatic decision making. We certainly agree with them that, “many trials would be better approached pragmatically.” We have included this paper in the discussion section.

 However, unless I have misunderstood what the authors are doing (which I do not exclude but in that case they should clarify this) the simulation is not a valid proof of what they claim, even for the example chosen.

 RESPONSE: Our aim was to illustrate the principles of this approach using a case study rather than provide “proof” that this approach is always better. We have changed the title to reflect this.

 The problem is the following. By simulating from the particular trial results, they are simulating from a universe in which the treatment is effective. This would be true even if the results from the TEXT ME trial had not been 'significant'. It is true of any trial in which the observed results favour the intervention. To see this consider that valid statistical analyses will typically have type I error rates in excess of a chosen nominal value if the mean under the intervention is greater (assuming high values are good) than the mean in the control group in the population in question. Provided that the type I error rate is controlled when this is not the case, this is a desirable property of such tests.

 RESPONSE: We agree, although our approach includes the changes to costs from implementing the TEXT ME intervention, so the mean difference also has to also be practically significant in order to recover these costs.

 Usually, the population in question is taken to be the population of all possible randomisations of the patients. Here, the authors sampled without replacement from the population. The population from which they are sampling is the population of results in the full TEXT ME trial. However, this is a population in which on average the results were better for the intervention.

 RESPONSE: We sampled with replacement. Using our approach, the group means were not always greater in the intervention group. For the primary outcome of LDL cholesterol, the mean was worse in the TEXT ME sample compared with usual care for around 22% of simulations when using the smallest sample size of 100. The mean difference in the secondary outcome of systolic blood pressure was stronger in the original data, and in simulations the mean in the TEXT ME sample was always lower (better) compared with the usual care group.

 Hindsight is an exact science but those making practical healthcare decisions are involved in the quite different game of foresight and they need to know whether the decision they are about to make is a reasonable one. This requires their allowing for the possibility that the intervention is useless or even harmful. Thus a mixture of possible situations has to be considered: simulating only from the case where the intervention is beneficial is not adequate.

 RESPONSE: Our aim is to provide results that are useful for decision makers, including estimates of uncertainty about the decision.

 In fact the precise nature of the mixture envisaged can have a huge effect on the inferences. Recently, a number of authors have called for statistical standards of evidence to be modified in the opposite direction. For instance Benjamin et al.2  have suggested that the standard of p=0.005 should be adopted. David Colquhoun3 has proposed an even more stringent standard of P=0.001.  This flows from the particular approach to Bayesian hypothesis testing which places a lump of probability on no difference between treatments. (See my blog4 for a discussion.) In my opinion, these are not good suggestions for a number of reasons, including that such prior distributions are far too informative and that these authors implicitly assume, which is far from obviously the case, that the explanatory purpose of clinical trials is more important than the pragmatic one.

 RESPONSE: We agree and the tension of the explanatory versus pragmatic trial is a key motivation for this paper. These adjustments to the use of the p-value remain focused on the p-value and it how can inform decisions. These adjustments have been motivated by prior abuses and misinterpretations of the p-value, which is a prosaic statistic. Our approach aims to give decision makers, working under conditions of scarce resources, more meaningful statistics regarding changes to costs and health benefits.

 However, I agree entirely with the authors, that as soon as practical decision-making involving economics is involved, it is the value of information that is important. In this connection, I can recommend the work of Forster, Pertile and colleagues5,6. See also Burman et al. 7

 RESPONSE: Thanks for flagging these interesting papers.

 Thus, I think to make good their claim, the authors would, at the very least, need to simulate from a universe in which the intervention was not necessarily better than the control. Unless I have misunderstood, this was not the simulation they undertook.

 RESPONSE: We have added just such a simulation, which shows that when there’s no treatment benefit there is a positive cost from the intervention that is not outweighed by any quality of life benefit. The cost-effectiveness plot shows clear evidence against adopting the intervention. We have added the methods and results for this new simulation and include Figure 3.

 References

 1. Schwartz D, Lellouch J: Explanatory and pragmatic attitudes in therapeutical trials.J Chronic Dis. 1967; 20 (8): 637-48 PubMed Abstract

 2. Benjamin D, Berger J, Johannesson M, Nosek B, Wagenmakers E, Berk R, Bollen K, Brembs B, Brown L, Camerer C, Cesarini D, Chambers C, Clyde M, Cook T, De Boeck P, Dienes Z, Dreber A, Easwaran K, Efferson C, Fehr E, Fidler F, Field A, Forster M, George E, Gonzalez R, Goodman S, Green E, Green D, Greenwald A, Hadfield J, Hedges L, Held L, Hua Ho T, Hoijtink H, Hruschka D, Imai K, Imbens G, Ioannidis J, Jeon M, Jones J, Kirchler M, Laibson D, List J, Little R, Lupia A, Machery E, Maxwell S, McCarthy M, Moore D, Morgan S, Munafó M, Nakagawa S, Nyhan B, Parker T, Pericchi L, Perugini M, Rouder J, Rousseau J, Savalei V, Schönbrodt F, Sellke T, Sinclair B, Tingley D, Van Zandt T, Vazire S, Watts D, Winship C, Wolpert R, Xie Y, Young C, Zinman J, Johnson V: Redefine statistical significance. Nature Human Behaviour. 2018; 2 (1): 6-10 Publisher Full Text

 3. Colquhoun D: An investigation of the false discovery rate and the misinterpretation of p-values.R Soc Open Sci. 2014; 1 (3): 140216 PubMed Abstract | Publisher Full Text

 4. Senn SJ: Double Jeopardy: Judge Jeffreys upholds the law. 2015. Reference Source

 5. Pertile P, Forster M, Torre D: Optimal Bayesian sequential sampling rules for the economic evaluation of health technologies. Journal of the Royal Statistical Society: Series A (Statistics in Society). 2014; 177 (2): 419-438 Publisher Full Text

 6. Jobjörnsson S, Forster M, Pertile P, Burman CF: Late-stage pharmaceutical R&D and pricing policies under two-stage regulation. J Health Econ. 2016; 50: 298-311 PubMed Abstract | Publisher Full Text

 7. Burman C-F: Decision Analysis in Drug Development. In: Dmitrienko A, Chuang-Stein C, Agostino R, eds. Pharmaceutical Statistics Using SAS: A Practical Guide.Cary: SAS Institute. 2007. 385-428
